# Contemporary challenges in the management of advanced heart failure

**DOI:** 10.3325/cmj.2014.55.551

**Published:** 2014-12

**Authors:** Hrvoje Gašparović, Davor Miličić, Bojan Biočina

**Affiliations:** 1 Department of Cardiac Surgery, University Hospital Center Zagreb, University of Zagreb School of Medicine, Zagreb, Croatia; 2 Department of Cardiology, University Hospital Center Zagreb, University of Zagreb School of Medicine, Zagreb, Croatia

Heart failure (HF) is a public health concern with an immense effect on the utilization of precious and finite health care resources. More than 23 million people worldwide have been affected by symptoms of HF (1). The equivocal criteria for diagnosing HF have led to variable projections of its true incidence, but its impact and frequency grant it the status of a modern-day epidemic (1). Its prevalence continues to follow an upward trend, as it parallels the economic growth and progressive aging of a community. The syndrome of HF is multifaceted in origin, and encompasses a wide range of underlying clinical entities that all result in pronounced morbidity and mortality.

The changing demographic profiles of the present-day HF populations underscore the importance of pursuing a multidisciplinary approach that will vigilantly address not only the cardiac pathology, but also the numerous accompanying comorbidities. Mortality burden of heart failure is comparable to or greater than that of malignancies such as melanoma, breast, kidney, and colorectal cancers (2). Promotion of campaigns that facilitate the perception of HF as an ominous disease should, therefore, be advocated.

The convergence of increasingly more effective acute HF therapies and the adverse downstream effects of modern-day lifestyles will likely lead to a further increase in chronic HF patients.

In this issue of the *Croatian Medical Journal*, multiple venues of advanced HF treatment are reviewed (3-10). The issue includes scholarly articles portraying some of the fundamental facts pertaining to heart failure and its management, while also focusing on specific clinical problems.

Although heart transplantation has marginal epidemiological gain, it remains the benchmark against which all other modes of management must be compared. The excellent 10-year survival of heart transplantation recipients reinforces its position as the gold standard in the management of end-stage HF (11). While over the past decades substantial advances in HF pharmacotherapy, biventricular pacing, and mechanical circulatory assistance have been made, the superiority of long term outcomes of heart transplantation remains unchallenged. This issue of the *CMJ* contains a comprehensive report on pre-transplant and perioperative predictors of heart transplantation outcomes (3). Multiple issues associated with organ transplantations such as immunosuppression, rejection, infections, and neoplastic disease act in concert to compromise the long-term outcomes. Furthermore, cardiac allograft vasculopathy exerts an important impact on long term survival of transplant recipients. Its insidious and progressive nature warrants a comprehensive analysis, which was conducted in one of the articles in this issue (4)

The principal limitation of heart transplantation, however, stems from a continued lack of available organs. This discrepancy between limited donor availability and high organ demand provides a fertile ground for the implementation of implantable ventricular assist devices. High-quality survival supremacy of mechanical circulatory assistance over optimal medical therapy among patients with end-stage HF is juxtaposed to the high incidence of complications that accompany this technology (5). Device-related infections, bleeding, and stroke are all potentially catastrophic to the patient, and this is highlighted among LVAD recipients in whom a destination strategy is pursued. With only 30% of long-term LVAD recipients being free from any adverse event at 1 year post-implantation there is clearly room for improvement in both device design and postoperative management (12). Also, from a clinical standpoint, patient selection and timing of implantation remain paramount.

Among many challenges that exert an important impact on outcomes in this patient population, clearly pivotal is the management of the failing right ventricle. Options for long-term support of the failing right ventricle have not followed the rapid evolution seen in the LVAD arena. A common clinical scenario is unmasking of incipient right ventricular failure after LVAD implantation. Multiple laboratory, clinical, and echocardiographic parameters have been employed in the effort to identify LVAD patients in whom right ventricular failure will occur, but an effective predictive system is still not available. Nonetheless, this issue presents a summary of available scoring systems and their individual performances (6).

While the focus of the medical and industrial communities on long-term implantable devices is certainly warranted, one should also appreciate the progress made in the domain of supporting the circulation during acute cardiac failure (5,7,8). The modern heart failure specialist requires considerable expertise in navigating through the ever-expanding portfolio of available devices. This issue of the *CMJ* provides a roadmap that aims to expedite this process.

The revolutionary paradigm shift from pulsatile to continuous-flow ventricular assist devices allowed for their miniaturization, thereby rapidly expanding their clinical applicability. Also, the benefits of this technology have been underscored by improved durability and a lower incidence of adverse events. The clear advantages of novel devices notwithstanding, adverse sequelae of long-term exposure to non-pulsatile blood flow have been identified. An article in this issue (9) reviews the evidence on the link between non-pulsatile flow and several unfavorable clinical outcomes, such as adverse aortic valve remodeling, less efficient blood flow at the capillary level, and acquired von Willebrand’s syndrome.

Reducing the high thromboembolic burden associated with HF may become another therapeutic goal in this challenging patient population. The current evidence on antithrombotic agents in HF patients, however, does not support their wider implementation (10).

In order to put the contemporary management strategies for advanced HF into perspective one has to contrast their unequivocal efficacy with their high financial and logistical burdens. Balancing these opposing forces requires finesse, and is pivotal to optimizing the results of HF management programs. Furthermore, equality of opportunity for all patients in the utilization of these finite resources should be fostered. The purpose of this theme issue is to disseminate information related to heart failure management, while providing insight into the associated challenges.
